# Using Persuasive Technology to Increase Physical Activity in People With Chronic Obstructive Pulmonary Disease by Encouraging Regular Walking: A Mixed-Methods Study Exploring Opinions and Preferences

**DOI:** 10.2196/jmir.6616

**Published:** 2017-04-20

**Authors:** Yvonne Kiera Bartlett, Thomas L Webb, Mark S Hawley

**Affiliations:** ^1^ Manchester Centre for Health Psychology Manchester Academic Health Science Centre, The School of Health Sciences University of Manchester Manchester United Kingdom; ^2^ Department of Psychology University of Sheffield Sheffield United Kingdom; ^3^ Centre for Assistive Technology and Connected Healthcare and School of Health and Related Research University of Sheffield Sheffield United Kingdom

**Keywords:** persuasive technology, chronic obstructive pulmonary disease, physical activity, walking, mHealth, mobile apps

## Abstract

**Background:**

People with chronic obstructive pulmonary disease (PwCOPD) often experience breathlessness and fatigue, making physical activity challenging. Although many persuasive technologies (such as mobile phone apps) have been designed to support physical activity among members of the general population, current technologies aimed at PwCOPD are underdeveloped and only use a limited range of persuasive technology design principles.

**Objective:**

The aim of this study was to explore how acceptable different persuasive technology design principles were considered to be in supporting and encouraging physical activity among PwCOPD.

**Methods:**

Three prototypes for mobile apps using different persuasive technology design principles as defined by the persuasive systems design (PSD) model—namely, dialogue support, primary task support, and social support—were developed. Opinions of these prototypes were explored through 28 interviews with PwCOPD, carers, and the health care professionals (HCPs) involved in their care and questionnaires completed by 87 PwCOPD. Participants also ranked how likely individual techniques (eg, competition) would be to convince them to use a technology designed to support physical activity. Data were analyzed using framework analysis, Friedman tests, and Wilcoxon signed rank tests and a convergent mixed methods design was used to integrate findings.

**Results:**

The prototypes for mobile apps were received positively by participants. The prototype that used a dialogue support approach was identified as the most likely to be used or recommended by those interviewed, and was perceived as more persuasive than both of the other prototypes (*Z*=−3.06, *P*=.002; *Z*=−5.50, *P*<.001) by those who completed the questionnaire. PwCOPD identified dialogue support and primary task support techniques as more likely to convince them to use a technology than social support techniques (*Z*=−5.00, *P*<.001; *Z*=−4.92, *P*<.001, respectively). Opinions of social support techniques such as competition and collaboration were divided.

**Conclusions:**

Dialogue support and primary task support approaches are considered to be both acceptable and likely to be persuasive by PwCOPD, carers, and HCPs. In the future, these approaches should be considered when designing apps to encourage physical activity by PwCOPD.

## Introduction

The term “persuasive technology” describes “any interactive computing system designed to change people’s attitudes and behaviors” (p.1; [1]). There are estimated to be over 40,000 mobile phone apps that aim to persuade users to change health behaviors such as physical activity, diet, and smoking [2]. However, despite a rising number of publications in the area [3], content analysis of existing apps reveal that they currently make little use of theories on behavior change or persuasive technology and include little evidence-based content [4-8]. As a consequence, there have been multiple calls for people to make better use of theory and evidence when designing apps intended to promote behavior change [9-11].

The persuasive systems design (PSD) model was developed to provide a framework for the design and evaluation of persuasive technologies [12]. Expanding on Fogg’s (2003) conceptualization of persuasive technology [1], Oinas-Kukkonen and Harjumaa (2009) identified 28 techniques that can be used to design persuasive systems. These are organized into four persuasive design principles, or approaches, to persuasion: (1) techniques that help the user to carry out the target behavior (termed *primary task support*), (2) techniques that motivate the user through feedback and interaction with the technology (termed *dialogue support*), (3) techniques that leverage social influence (termed *social support*), and finally, (4) techniques that make the system appear credible to the user (termed *credibility support*) [12]. Table 1 provides examples of techniques associated with each of these design principles. Initial evidence suggests that including dialogue support techniques in technology-based interventions may increase adherence [13], the extent to which the intervention is perceived to be persuasive and, through increasing people’s motivation to use the intervention, effect actual use of the persuasive system [14]. Despite this evidence, however, theoretical approaches to designing the interactive elements of persuasive technologies remain underused [13,15].

**Table 1:** Examples of persuasive technology techniques by design principle [12].

Design principle	Example persuasive technology technique	Description
Primary task support	Reduction	A system that reduces complex behavior into simple tasks helps users perform the target behavior, and may increase the cost-benefit ratio of a behavior.
Dialogue support	Social role	If a system adopts a social role, users will be more likely to use it for persuasive purposes.
Social support	Competition	A system can motivate users to adopt a target attitude or behavior by leveraging human beings’ natural drive to compete.
Credibility support	Trustworthiness	A system that is viewed as trustworthy will have increased powers of persuasion

### Increasing Physical Activity in PwCOPD

Chronic obstructive pulmonary disease (COPD) is an umbrella term for a number of lung diseases such as chronic emphysema and chronic bronchitis. In 2013, COPD was the third most common cause of death in the United States [16], and the World Health Organization estimates that by 2030 it may become the third most common cause of death worldwide [17]. On average, each year, treating COPD typically costs the US health care system US $30 billion and the UK National Health Service (NHS) around £800 million [16-18]. PwCOPD experience symptoms such as breathlessness on exertion, muscle weakness, increased sputum production, and a chronic cough. In the short-term, these symptoms can reduce people's ability to complete daily activities and reduce quality of life [19] and, in the long-term, these symptoms can lead to hospitalization and respiratory failure [20]. PwCOPD can enter a negative cycle as their symptoms make it harder to remain active, and the less active they are, the worse their symptoms become [21,22]. Indeed, evidence suggests that physical inactivity is associated with higher numbers of hospital admissions, exacerbations, and mortality in PwCOPD [23-25]. Currently, it is recommended that stable COPD is managed with a combination of medications and pulmonary rehabilitation (PR) [26,27]. PR has been shown to increase people’s capacity for exercise and their health-related quality of life [28]. However, studies have suggested that completing a course of PR does not necessarily increase levels of physical activity [29,30]. This finding suggests that although structured rehabilitation can increase the ability to perform physical activity, additional support may be needed to integrate physical activity into everyday life. Walking is a low-intensity, free physical activity that does not require specialist equipment or tuition. Regular walking after PR has been associated with higher quality of life and health-related quality of life [31], and increases in distance walked daily and daily step count have been found to predict fewer acute exacerbations in people with COPD [32,33]. Encouraging daily walking could therefore be an effective way to help increase physical activity in everyday life.

### Using Technology to Support Physical Activity in PwCOPD

Previous research on how technology can be used to support physical activity in PwCOPD has explored how both Internet and mobile phone-based technologies can encourage and support physical activity among PwCOPD, either alone or in conjunction with counseling [34-36]. The findings indicate that technology-based interventions are usually acceptable to PwCOPD and, although the studies thus far are mostly feasibility and pilot studies, early findings suggest that technology-based interventions have the potential to increase levels of physical activity in this population [36,37]. Indeed, to date, randomized trials have shown improvements in physical activity up to 3 months [38] and daily step count up to 4 months [39]. However, it should be noted that, in the latter study, engagement with the intervention had decreased by 12 months, and daily step count was not significantly different from baseline at this point [39].

An additional problem (which may contribute to the limited efficacy) is that, to date, interventions have tended to rely on a relatively small pool of techniques for promoting changes in behavior, namely, self-monitoring, providing feedback, motivational suggestions, and goal-setting [34-39]. As a result, it is not currently known how acceptable a wider range of persuasive technology techniques would be and what design principles would be most attractive and persuasive to PwCOPD, their carers, and the health care professionals (HCPs) involved in their care.

### Aims

This research therefore sought to explore how acceptable and persuasive technologies following different persuasive design principles are in increasing physical activity through encouraging daily walking among people with chronic obstructive pulmonary disease (PwCOPD). Our aim was to inform the choice of design principles and specific persuasive techniques in the design of an app that could be used to encourage physical activity in this population. To achieve this, we designed three prototype apps and used them to investigate the following research questions:

RQ1: What are the opinions and preferences of PwCOPD, their carers, and HCPs involved in their care toward systems using different persuasive technology approaches?

RQ2: Which individual techniques, or design principles, are perceived to be most persuasive?

## Methods

### Design

A convergent mixed methods design was used to assess the opinions and preferences of PwCOPD, carers, and HCPs toward persuasive technology [40]. Ethical approval for the study was granted by the ethics committee at the University of Sheffield and permission to recruit was granted by both the NHS (for HCPs) and the British Lung Foundation.

### Participant Recruitment

Participants for the interviews were recruited through four British Lung Foundation Breathe Easy support groups in South Yorkshire (PwCOPD and carers) and from an NHS service specializing in care for PwCOPD (HCPs). Following approval from the moderators of the groups and a manager at the NHS service, potential participants were given information about the project. Anyone who was interested then contacted the researchers to participate. All participants provided informed consent.

A second sample of PwCOPD was invited to complete a questionnaire, either through a website or by post. A letter was sent to 140 Breathe Easy support groups (excluding those in South Yorkshire), which contained a link for Web-based completion and a number to call if participants preferred to receive a paper copy of the questionnaire. In addition, 34 moderators of online support groups for PwCOPD were contacted and 6 agreed to post a link to the questionnaire on their websites. All participants provided either written or electronic consent and no incentives were provided for participation.

### Materials

#### Prototypes

Three “medium-fidelity” prototypes were created to show how the screens within each system might look and to describe how users might navigate through the system. Medium-fidelity prototypes present the layout and content of each screen as it would look; not a sketch, as would be expected for a low-fidelity prototype; however, also they are not interactive, as would be expected for a high-fidelity prototype [41]. Navigation through the screens was shown with an arrow indicating which button should be pressed to move to the next screen. The prototypes were presented on a screen or the screenshots were presented on paper. Each prototype focused on a different persuasive technology design principle as delineated by the PSD model: dialogue support, primary task support, and social support. It was decided not to develop a prototype describing credibility support as the research was being conducted through a University and this may have inferred some credibility. It would therefore be difficult to know whether to attribute credibility to the persuasive technology technique or techniques or the context of the research. Each prototype used the same behavior change techniques associated with control theory [42]; namely, prompt intention formation, prompt specific goal setting, prompt self-monitoring of behavior, receive feedback, and prompt review of behavior [42,43]. All prototypes were designed to monitor a daily walk, in addition, certain persuasive technology techniques were present in all three prototypes such as self-monitoring, tailoring, and reduction [12]. The prototypes were further informed by looking at the most popular physical activity apps available for Android, iPhone, and Windows phone at the time of development to see how techniques were operationalized in popular apps designed to promote physical activity. The look and feel of the prototypes was standardized as far as possible, with each using the same font, font sizes, button design, and color scheme.

##### Prototype 1: Virtual Coach System

**Figure 1:**
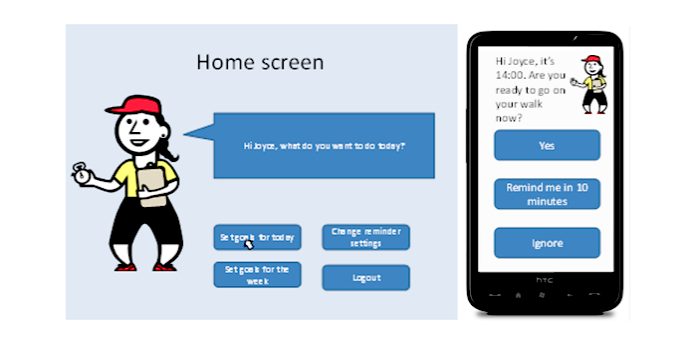
Screenshots from the virtual coach prototype showing the homepage and a reminder.

This prototype used a dialogue support approach that was designed to encourage interaction between the user and the system. In this prototype, the virtual coach represented by a static picture, used the name of the user (“Joyce”) to personalize the system and encourage interaction by taking a social role. The coach led the user through progressive goals. Although there was the option to change the goals, suggestions were made by the coach. The user could then choose to receive reminders to complete the activity (see Figure 1). The prototype explained that, while the user is walking, they would have the option to receive audio encouragement from the coach (in the form of recorded messages telling the user how many minutes they have been walking, or when they are halfway to their goal). Feedback would be presented as a graph, accompanied by praise and encouragement from the virtual coach. The prototype also outlined a suggested exercise plan with daily walking goals that increased to reach an overall goal (walking for 30 minutes).

##### Prototype 2: Music and Maps System

**Figure 2:**
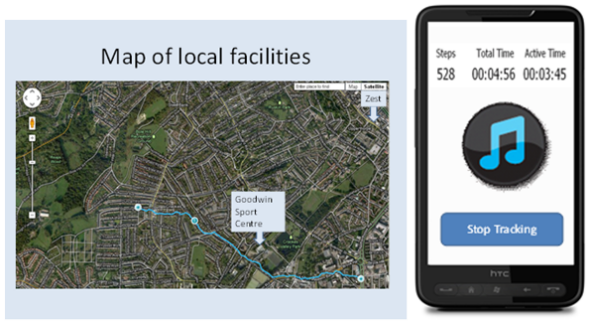
Screenshots from the music and maps prototype showing the map feedback and playing music.

This prototype used a primary task support approach that was intended to help the user to achieve their daily walking goal. The prototype was based on the format used by many of the existing apps designed to promote physical activity. In this prototype, participants could see that the user could set goals and track their activity using their mobile phone. It was explained that, while walking, the user could choose music to listen to. Following the walk, feedback would be offered on a satellite map, as a summary table, or on a calendar (with activity levels shown for each day). It was further explained that local exercise facilities would be highlighted on the map (see Figure 2).

##### Prototype 3: Online Community System

**Figure 3:**
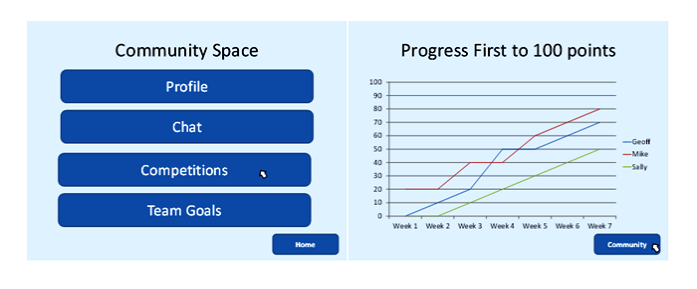
Screenshots from the online community prototype showing the community space and competition graph.

This prototype used a social support approach and was based on the idea of building a community of like-minded users to support increases in physical activity (see Figure 3). The prototype described how the app would provide a mechanism for computer-mediated communication between peers, while encouraging interaction through competitions and collaborations. The prototype showed that users could track their activity using a mobile phone, and share information with other users. It was explained that points would be given when users achieved their goals (the details of the goal completed would not be shared) and that there was the potential for users to earn both virtual (stars or trophies on their profile) and “real-world” rewards (either through vouchers or donating money to charity).

#### Interview Guide and Questionnaire

Both the questionnaire and the interview schedule followed the same basic structure. PwCOPD were first asked background questions related to COPD and their current level of physical activity. All participants were asked about their use of technology and whether they had any previous experience with persuasive technology. This was followed by a presentation of each of the three prototypes with an explanation to describe how the user might navigate through the system, following which participants’ opinions were sought before the next prototype was presented. Once participants had expressed their opinions on all three prototypes, their overall opinion of using persuasive technology to support and encourage physical activity within PwCOPD, and preferences for specific prototypes as well as techniques and features within the prototypes, were sought.

The interview was piloted with a person with COPD. As this participant reported no problems in understanding the interview material, and the timing was appropriate, it was decided to include the data from this participant within the main analysis. The Web-based questionnaire was piloted to ensure that all of the branch questions were working effectively, and that the prototypes were displayed appropriately. A paper version of the questionnaire was piloted with 4 people aged 31-60 years in order to test whether the branch questions were clear, and to establish how long it typically took people to complete the questionnaire. Time taken ranged from 15 to 30 minutes. No problems were reported with the branch questions. However, we did reverse the items identified as negative items in the measures section (so a high score would indicate a more positive response) and correct an error in the information section. As those who piloted the questionnaire did not have COPD, their data were not used in the analysis.

#### Questionnaire Measures

The questionnaire was divided into four sections:

Section 1 “Questions about you” included questions about demographics (eg, age and gender), how long the participant had been diagnosed with COPD, and the Medical Research Council (MRC) breathlessness scale [44,45] which comprises 5 statements that ask participants to grade their current experience of breathlessness from “Not troubled by breathlessness except on strenuous exercise” (MRC grade 1) to “Too breathless to leave the house or breathless when dressing or undressing” (MRC grade 5).

Section 2, “Questions about physical activity” asked participants to estimate the number of minutes per week that they engaged in light, moderate, and vigorous intensity activity (definitions were provided of each of these, based on the information in [46]).

Section 3, “Questions about technology” was developed for this study and included questions related to computer and mobile phone ownership and use. Participants were also asked if they had “heard of or seen,” “ever used,” “still use,” or “would consider using” “any technology (ie, on the computer, on the Internet, or mobile phone) that is designed to try and help change people’s behaviors, for example, increasing exercise, encouraging healthy eating, or stopping smoking.”

Finally, Section 4 assessed participants’ opinion of each prototype using 8 items, of which 4 items were translated from the *perceived persuasiveness* measure [14]; that is, participants were asked how much they agreed that each prototype “was interesting,” “would have an influence on me,” “is personally relevant to me,” and “makes me think about my physical activity.” An additional 4 items were added to assess the extent to which participants expected to enjoy using the technology (“I would not enjoy using this system” [negative item]) and how effective they thought it might be (“I think the system would be useful in increasing my physical activity,” “If I wanted to increased my activity levels, I would not use this system” [negative item], and “this system makes me want to increase my physical activity”). All the items were answered on a 7-point Likert scale from “strongly agree” to “strongly disagree,” and the negative items identified above were reverse-scored, and thus a higher score indicates a more positive response. Reliability across the 8 items was assessed using Cronbach alpha and was found to be high for each prototype (virtual coach, Cronbach alpha=.93; music and maps, Cronbach alpha=.93; and online community, Cronbach alpha=.95) [47]. Therefore, the 8 items were summed to create a single scale score representing how persuasive each prototype was deemed to be. Participants were also given a full list of the features across the prototypes and asked to rank the top five that they believed might convince them to use the technology and also to indicate with an “x” any features that would definitely not convince them to use the technology.

In the interview, preferences were elicited by asking participants to identify which prototype they would use (or recommend to others in the case of HCPs). To allow comparison with the questionnaire data, these responses were coded [48] such that if a clear choice was made, then the prototype or feedback screen was given a score of 1; whereas, if a participant reported that they would choose a combination of two prototypes or that two were equally favored, each was given a score of 0.5.

### Data Analysis

Framework analysis [49] was used to analyze the interview data in Nvivo 9 (QSR International Pty Ltd). The questionnaire data were analyzed with SPSS 17.0 (SPSS Inc). Preferences for features were calculated using the ranked score given to the individual techniques (recoded such that a ranking of 1 gave the highest score). Individual techniques were grouped according to the PSD (primary task support, dialogue support, or social support) [12] and the Friedman and Wilcoxon signed rank test were used to identify differences in ranking between the principles.

The data from the interviews and questionnaires were analyzed separately, and then an integration matrix was designed to compare the two strands of data [40,48,50,51]. The integrated findings are presented under thematic headings in Results section below.

## Results

### Sample Characteristics

Table 2 describes the characteristics of the sample. In total, 23 interviews were conducted; 11 with PwCOPD on their own, 5 with PwCOPD and their carers, and 7 with HCPs (providing a total N of 28). Questionnaires were returned by 121 PwCOPD; however, 34 were excluded due to missing data. The analyses reported below are therefore based on those who rated how persuasive PwCOPD found each prototype (n=87). Mild COPD was underrepresented in both samples: The modal MRC breathlessness grade reported by PwCOPD who completed the questionnaire was 4, and 69% (11/16) of PwCOPD who were interviewed reported needing help when walking outside.

Most of the participants who were interviewed had a mobile phone (although some rarely used it). However, very few of these participants had ever heard of or used any form of persuasive technology. The participants who responded to the questionnaire seemed to be more familiar with technology, with 91% (79/87) of the participants having a mobile phone, and 52% (41/79) using it at least daily. Furthermore, 46% (40/87) of the participants had heard of persuasive technology, and 63% (46/73) reported that they would consider using persuasive technology.

**Table 2:** Participant characteristics.

Characteristics		Interview (*n*=28)	Questionnaire (*n*=87)
Mean age (SD^a^)		70.8 (8.3)^b^	64.0 (8.5)
% of female participants		16 (57%)	59 (69%)^c^
Nationality UK		28 (100%)	58 (67%)
**MRC^d^breathlessness grade**			
	1		5 (6%)
	2		21 (24%)
	3		19 (22%)
	4		28 (32%)
	5		14 (16%)
≥150 min moderate activity per week^e^			39 (65%)

^a^SD: standard deviation.

^b^Only PwCOPD, n=16.

^c^n=85.

^d^MRC: Medical Research Council.

^e^n=60.

### Participants’ Opinions of the Prototypes and Preferences

#### Prototype 1: The Virtual Coach System

Participants who were interviewed tended to think that encouragement from the virtual coach would be motivating, which could indicate that the prototype was successful in describing a system that could fulfill a social role:

You are motivated when you’re encouraged.

PwCOPD, female, aged 65 years

The virtual coach system was thought to be good for people who were more mobile and those living alone. Carers described similar sentiments, namely, that the virtual coach system would be good for people who are mobile, on their own, and able to use the technology.

If they can manage the technology... And if it was somebody on their own who needed (it).

Carer, female, aged 75

Participants’ reasons for deeming the virtual coach system as suitable for someone on their own were that if a carer was motivating the person with COPD, then there would be no need to duplicate this role through technology, again suggesting that participants viewed this form of technology as fulfilling a social role. HCPs were very positive about the prototype, although they felt that the novelty of a virtual coach may wear off and that not everyone would understand the technology. They therefore felt that there should be an opportunity to simplify the virtual coach if the full system was deemed too complex. HCPs also tended to think that the technology suggesting goals would be useful.

I think that’s what a lot of people need because if you’re just doing it yourself, you just switch off and say oh, another day.

HCP, female

**Figure 4:**
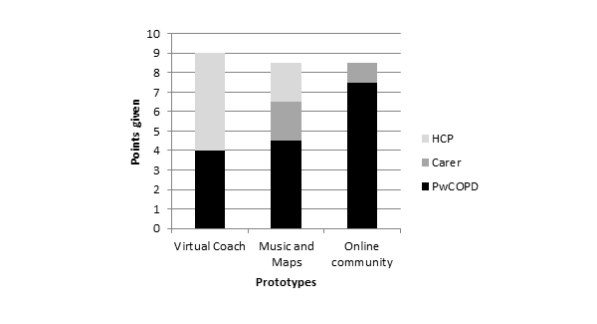
Preference for prototypes expressed during the interviews. HCP: health care professional; PwCOPD: people with chronic obstructive pulmonary disease.

When asked which system they would use (or recommend for use) the virtual coach system was given the highest score by HCPs, but it was not scored as highly as the other prototypes by PwCOPD or their carers (see Figure 4).

**Figure 5:**
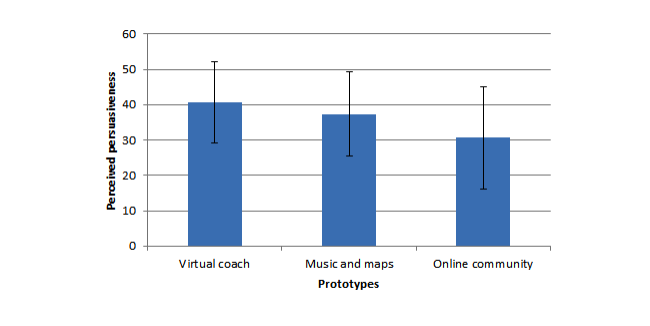
Perceived persuasiveness of each prototype (questionnaire data).

Participants who completed the questionnaire perceived the virtual coach prototype to be the most persuasive (mean 40.71, SD 11.46). A Friedman test identified a significant main effect of prototype (χ^2^_2_=28.1, *P*<.001), and a Wilcoxon signed rank test (with Bonferroni correction applied) found significant differences between the virtual coach and the music and maps prototypes (*Z*=−3.06, *P*=.002) and between the virtual coach and the online community prototypes (*Z*=−5.50, *P*<.001; see Figure 5).

In summary, both samples agreed that the virtual coach system could be persuasive, although the PwCOPD who were interviewed were less positive about the prototype than PwCOPD who answered the questionnaire and the HCPs and carers who were interviewed.

#### Prototype 2: The Music and Maps System

When interviewed, both PwCOPD and HCPs mentioned that the maps and the summary information provided in the music and maps prototype seemed like it might be interesting, but that it may not be sufficient to promote physical activity. Participants felt that goals that were suggested by the system may be a useful addition to help to persuade people to perform the activity suggested.

I do like the maps and where you’ve walked, and tracked and I think that’s good, because look I’ve done that, I might do a bit more tomorrow.

PwCOPD female, aged 70 years

I would, ideally, like a combination of both, in that if you want the system to suggest goals then it can do but if you’re quite happy with setting your own goals and you know what you want to achieve then it sort of takes a more stand back approach and lets you do it basically.

HCP, female

Again, the technology described in the music and maps prototype was thought to be better for people who were relatively mobile as the feedback would likely be more interesting if the person was walking further. Some PwCOPD mentioned that this system would not provide them with any useful information if they either walked the same routes or walked very little outside.

The idea of walking with music divided opinion, with some participants feeling that music was useful as a distraction while exercising, whereas others thought that it would be dangerous to walk while using headphones:

There are an awful lot of accidents caused by...walking along, their head’s in the clouds their big bopping through their ears...I personally think it’s not a good idea.

PwCOPD, male, aged 73 years

Some PwCOPD, HCPs, and carers indicated that they may use (or recommend that those they care for use) the music and maps prototype (see Figure 4). The PwCOPD who completed the questionnaire rated this prototype as less persuasive (mean 37.40, SD 11.85) than the virtual coach prototype (*Z*=−3.06, *P*=.002), but more persuasive than the online community prototype (*Z*=−3.82, *P*<.001) (see Figure 5).

#### Prototype 3: The Online Community System

Participants tended to think that an appreciation of the social components of this prototype would depend on the user’s personality; that is, it may appeal to some but not to others. One user had previously had a negative experience with an online support group and therefore said that they would not use one again; another remarked that the success of online communities depended on who else was using the website:

I’m a person person rather than a computer person. So for me, in my age group I have doubts, the younger end...they’re into Facebook, they’re into Twitter and whatever. I’m not.

PwCOPD, male, aged 74 years

Participants who liked the prototype describing the online community appreciated the potential for competition and for communicating with people who were going through similar experiences. Some PwCOPD felt that competition would motivate them and likened it to other competitive activities that they enjoyed like playing cards or quizzes:

It encourages you to do it both for your own sake and for the competition.

PwCOPD, male, aged 76 years

Some participants felt that an online community would be better for those who are more mobile; whereas, other participants thought that people who could not do much activity would be more likely to use such technology. HCPs tended to support the latter view, stating that the online community would be the best for people who cannot go out, but that the approach would only work if the user themselves chose it. One HCP said that it would be hard for her to suggest this system to PwCOPD as she did not like it herself.

Others felt that incorporating competition might promote an unhealthy desire to win and, relatedly, that losing may have a detrimental effect on the user’s feelings; or that being in competition was not in keeping with the purpose of this technology (which is to promote the self-management of COPD, and ultimately to feel better):

Is making it competitive taking the idea away from what you’re actually doing it for?

PwCOPD, male, aged 68 years

Some participants felt that their opinion of the prototype describing the online community system may be influenced by their own competitive nature; some described that being competitive would make losing harder, whereas others felt that being a competitive person would encourage them to try more. HCPs made the point that they try to discourage competition between people during PR, as it can result in people over-exerting themselves or feeling disheartened. When the idea that the actual goal (ie, how many minutes) would not be revealed to other users was reiterated, some HCPs changed their minds and became more supportive of the idea, whereas others felt that having hidden goals might encourage cheating and that the points would not necessarily go to the right people. One HCP felt that using persuasive technology to make any comparison between users would be inappropriate:

We try and avoid encouraging that sort of behavior, erm, and I’m quite a competitive person and you know the whole first person to get to 500 points and I would be, and I know this sounds really bad but I would be really inclined to decrease the amount of activity that I did to get my points quicker to beat someone.

HCP, female

When participants who were interviewed were asked which persuasive technology they would be most likely to use, the prototype describing the online community was chosen more often than the other two prototypes by PwCOPD. It was chosen by a few carers but not by any HCPs (see Figure 4). Respondents to the questionnaire rated the online community prototype as significantly less persuasive than the prototypes describing the music and maps and the virtual coach (mean 30.67, SD 14.52; *Z*=−5.50, *P*<.001 and *Z*=−3.82, *P*<.001, respectively; see Figure 5).

### Opinions of Individual Features and Persuasive Technology Techniques

**Figure 6:**
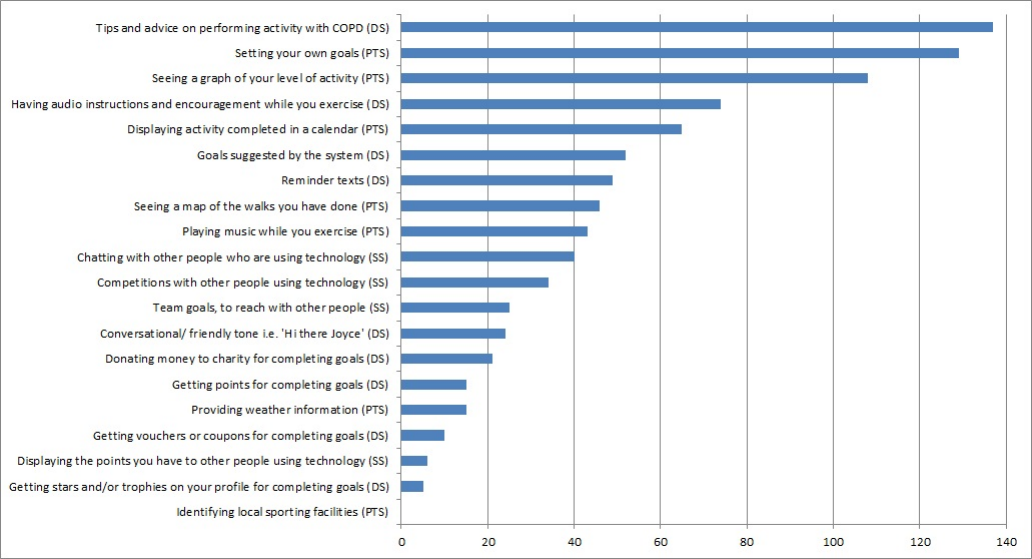
Sum of ranks given to individual techniques and features. Principles of the persuasive systems design model they relate to is indicated in brackets. PTS: primary task support; DS: dialogue support; SS: social support; COPD: chronic obstructive pulmonary disease.

Among the respondents to the questionnaire that ranked at least 5 features of the persuasive technologies (n=54), scores were reversed so that a high ranking was associated with a high score. Figure 6 shows how participants ranked the different features. The feature that was ranked as the most likely to convince participants to use the technology was “Tips and advice on performing activity with COPD.”

The features were then grouped according to the element of the PSD model that they addressed independently of the prototype they were presented within, namely, primary task support, dialogue support, or social support. A Friedman test identified a significant difference between the persuasiveness of features associated with different elements of the PSD model (χ^2^_2_=33.0, *P*<.001), and a Wilcoxon signed rank test (with Bonferroni correction applied) found that features associated with primary task support were rated significantly more likely to convince PwCOPD to use the technology (mean 7.52, SD 4.63) than those associated with social support (mean 1.94, SD 2.97; *Z*=−5.00, *P*<.001). Features associated with dialogue support were also rated significantly higher (mean 7.17, SD 5.53) than those associated with social support (*Z*=−4.92, *P*<.001). There was no significant difference between participants’ ratings of features associated with primary task support and those associated with dialogue support (*Z*=−.25, *P*=.80).

It was found that 32 participants (59% 32/54) also indicated that some features would definitely not convince them to use persuasive technology; the most commonly identified features being identifying local sporting facilities (n=23), getting stars or trophies on your profile for completing goals (n=22), and displaying the points that you have to other people who are using this technology (*n*=21).

## Discussion

The findings of this research suggest that persuasive technology techniques designed to encourage and support physical activity among PwCOPD were received relatively positively by PwCOPD, their carers, and HCPs involved in their care. Below, we discuss the findings in the context of the PSD model and other theoretical literature, as well as pointing to the practical implications of the findings.

### Principal Findings

The prototype based on the dialogue support principle that described a virtual coach was, overall, deemed most likely to be used or recommended, and it was also deemed to be the most persuasive. In addition, the techniques associated with the primary task support principle were ranked as most likely to convince participants to use a technology (although not significantly different to the techniques associated with dialogue support). The prototype based on the social support design principle that described the online community was the least likely to be recommended for use by HCPs and was also rated as the least persuasive by PwCOPD who completed the questionnaire.

The positive view of both dialogue support and primary task support expressed in this research suggests that these persuasive design principles and associated techniques are acceptable to PwCOPD and are likely to be used. In support of this idea, a review of apps designed to promote behavior change identified that primary task support elements are used most frequently in apps that target smoking, drinking, and weight loss, and the authors additionally suggest that dialogue support techniques might also be used to promote other behaviors [52]. The use of techniques associated with dialogue support has been shown to have a direct effect on how persuasive interventions are deemed to be, which in turn has been found to predict intentions to use and actual use of a website aimed to encourage weight loss and increase positive mood [14].

A recent systematic review found that self-monitoring was the most commonly used persuasive technology technique in apps that are designed to increase physical activity. Suggestions and praise were the most commonly used dialogue support techniques, whereas the technology taking a social role (also a dialogue support technique) was only used in a single paper [8]. Participants in this research felt that the social role provided by technology should not duplicate other forms of support. For example, those who received encouragement from a carer did not feel that they would also need it from a virtual coach. However, other participants reported that they would value encouragement from technology, perhaps because they did not receive it from other sources.

The competition element of the online community prototype divided opinion. Some PwCOPD felt that competition would be encouraging, whereas others felt that the danger of becoming disheartened was too great and that any system should aim to provide positive support and encouragement only. This opinion was echoed by the HCPs who were interviewed. Unlike rewards and maps, which were thought to be potential extras that might be ignored, HCPs seemed to recognize that competition could be persuasive, but felt that this approach to persuasion was inappropriate. Competition has been identified as a design element that is commonly used in games [53], and thus, could be considered a form of “gamification” (where game elements are used in nongame contexts such as health care [54]). Research that looks more broadly at the application of gamification has identified that the context that gamification is used in as well as characteristics of the user (eg, age) may influence the effectiveness of using game strategies to persuade [55,56], although little work thus far has explored the use of gamification in an older adult population [57].

One explanation for the divided opinion of competition in this study might be that different people anticipate experiencing different emotions as a result of competing. Some participants compared the competitive element provided by the online community to other competitive activities that they enjoyed such as playing cards or quizzes. These participants perhaps anticipated experiencing positive emotions when competing and viewed competition as fun. Other participants, however, may have feared failing and so reacted negatively to the prospect of competition. In the PSD model, competition is reported to leverage human beings’ “natural drive to compete” (p.495; [12]), and the inclusion of competitive elements has been found to have a positive effect on levels of physical activity interventions among healthy adults in some studies [8], as well as being frequently used in apps designed to promote weight loss [52]. It could be suggested, however, that the challenges that physical activity presents to PwCOPD means that competition is associated with stronger emotions. PwCOPD who appreciate the importance of physical activity may connect a failure to be active with a decline in their health, and therefore, the consequences of not succeeding in the competition may be viewed as more serious than they might be among healthy adults. Therefore, this hypothesis, as well as further exploration of the potential for other gamification strategies in the context of COPD care, warrants further consideration.

The findings suggest that a system that supports dialogue between the user and the technology alongside supporting the primary task (here, walking) to promote the self-regulation of physical activity is likely to be acceptable to PwCOPD and perceived as persuasive. Previous research indicates that these design principles are associated with adherence to Web-based health interventions [13] as well as intention to use and actual use of a Web-based intervention designed to promote healthy eating [14]. In contrast, the use of the social support design principle, while potentially engaging for some, is less likely to appeal to the majority of users. As discussed above, this is likely to be especially true for techniques that encourage any form of social comparison or competition. Further research should explore the use of persuasive technology techniques not only to promote both initial interest in the technology but also to support continued engagement. As this research also focused only on encouraging regular walking, it may also be helpful for future research to consider a wider range of physical activities that are suitable for PwCOPD (eg, wall push-ups) over the longer term. If PwCOPD are willing to engage with persuasive technology, then applications could also extend beyond promoting physical activity to other aspects of managing COPD such as promoting the use of breathing exercises, and providing relevant information.

### Conclusions

This research investigated the opinions of PwCOPD, their carers, and HCPs involved in their care toward the use of different forms of persuasive technology to support and encourage increases in physical activity among PwCOPD. Opinions of persuasive technology were on the whole positive; however, opinions depended on personal preferences and initial levels of capability and motivation to engage with both physical activity and technology. Our findings suggested that a prototype describing a virtual coach designed to support interactions between the user and the technology was the most popular, and that techniques related to both supporting dialogue and primary task support were better supported by participants than those related to social support. We therefore recommend that future research integrate dialogue and primary support techniques into apps for PwCOPD and build on these findings to further explore how persuasive technology can be used to engage and meet the needs of this population.

## Abbreviations

COPD: chronic obstructive pulmonary disease
HCP: health care professional
MRC: Medical Research Council
NHS: National Health Service
PR: pulmonary rehabilitation
PSD: persuasive systems design
PwCOPD: people with chronic obstructive pulmonary disease
